# Control of Prosthetic Hands via the Peripheral Nervous System

**DOI:** 10.3389/fnins.2016.00116

**Published:** 2016-04-08

**Authors:** Anna Lisa Ciancio, Francesca Cordella, Roberto Barone, Rocco Antonio Romeo, Alberto Dellacasa Bellingegni, Rinaldo Sacchetti, Angelo Davalli, Giovanni Di Pino, Federico Ranieri, Vincenzo Di Lazzaro, Eugenio Guglielmelli, Loredana Zollo

**Affiliations:** ^1^Unit of Biomedical Robotics and Biomicrosystems, Department of Engineering, Università Campus Bio-Medico di RomaRoma, Italy; ^2^INAIL Centro ProtesiVigorso di Budrio, Italy; ^3^Institute of Neurology, Università Campus Bio-Medico di RomaRoma, Italy

**Keywords:** PNS-based prosthetic hand, motion control, grasping, manipulation, sensory feedback

## Abstract

This paper intends to provide a critical review of the literature on the technological issues on control and sensorization of hand prostheses interfacing with the Peripheral Nervous System (i.e., PNS), and their experimental validation on amputees. The study opens with an in-depth analysis of control solutions and sensorization features of research and commercially available prosthetic hands. Pros and cons of adopted technologies, signal processing techniques and motion control solutions are investigated. Special emphasis is then dedicated to the recent studies on the restoration of tactile perception in amputees through neural interfaces. The paper finally proposes a number of suggestions for designing the prosthetic system able to re-establish a bidirectional communication with the PNS and foster the prosthesis natural control.

## Introduction

The complex mechanical structure, the composite sensory system, and the bidirectional communication between hand and brain make the human hand the most complex organ of the human body after the brain.

In the intact subject, hand movements are produced by more than thirty individual muscles located within the hand (intrinsic muscles) or forearm (extrinsic muscles). Intrinsic muscles, innervated by terminal branches of median and ulnar nerves, are responsible for fine motor control of the individual fingers, while extrinsic muscles, mostly innervated by median and radial nerves, produce gross flexion/extension movements of the whole hand and forceful grip.

Proprioceptive and sensory feedback are of paramount importance in all the activities of daily living (Johansson and Flanagan, 2009; Saal and Bensmaia, 2015). Individuals with intact upper-limb and motor pathways, deprived of somatosensory feedback, are characterized by slow and clumsy movements (Sainburg et al., 1993) and experience the impossibility of attending to complex tasks, such as buttoning a jacket (Sainburg et al., 1995).

In physiological tactile sensation, there are four types of mechanoreceptors in glabrous skin that respond to mechanical pressure or distortion and send projections to the central nervous system via large-diameter nerve fibers. Merkel discs, innervated by slowly adapting type 1 (SA1) afferents, underlie the perception of form and roughness; they have small receptive fields and produce sustained responses to static stimulation. Ruffini endings, connected to slowly adapting type 2 (SA2) fibers, respond to skin stretch; they have large receptive fields and produce sustained responses to static stimulation. Meissner corpuscles, associated with rapidly adapting (RA) fibers, respond to low-frequency skin vibrations and movements across the skin; they have small receptive fields and produce transient responses at the onset and offset of stimulation. Pacinian corpuscles (PC), respond to high-frequency vibration; they have large receptive fields and produce transient responses (Saal and Bensmaia, 2014).

Transmission of sensory information from the hand to the brain relies on signal transduction at the skin surface and signal transmission to the brain cortex through first order and second order somatosensory neurons, via the dorsal-column medial lemniscal pathway. Restoration of afferent information in case of missing hand could thus be grounded on the substitution of the signal transduction system with artificial sensors on the prosthesis and the stimulation of peripheral nerves or higher order structures through neural interfaces (as detailed in Section Hand Sensorization).

The hand loss causes severe impairment for the amputee and can significantly reduce quality of life. The relevance of the upper-limb loss in the international scenario [4000 people per year in Italy^1^, (Cutti et al., 2012) and 340,000 people living with limb loss in USA (NLLIC, 2007)] motivates the flourishing research in the field of upper-limb prosthetics. In the last 70 years, there have been significant improvements in the upper limb prosthetic field thanks to the advancements in the technological field and in the surgical procedures. Prostheses are more and more conceived to reproduce aesthetical as well as functional features of the lost limb, thus fostering improvements in hand design, control and sensory feedback, in order to meet prosthetic user needs (Biddiss et al., 2007).

This paper intends to provide an exhaustive review of the literature on PNS-based control of hand prostheses taking into account both technical aspects regarding myoelectric hand control and hand sensorization, and features related to human experimentation for the restoration of the sensory feedback. Moreover it aims at defining, based on the reviewed literature, a number of suggestions regarding control system, force/tactile sensory system and restoration of the tactile sensation and outlining future perspectives in this field.

For clarification, with PNS-based control the control of prostheses through interfaces with the PNS is intended, including electromyographic (EMG) interfaces, measuring signal from the muscles, as well as neural (ENG) interfaces, measuring signal from the nerves. As in Navarro et al. (2005), in fact, the EMG interfaces are regarded as simple neural interfaces (Schultz and Kuiken, 2011) since the electric signals generated by the muscles are the result of movement commands generated in the motor cortex and propagated along the peripheral nerves.

The analysis of the studies on CNS-based control of artificial limbs (e.g., Hochberg et al., 2006; Cloutier and Yang, 2013; Thakor, 2014) is outside the scope of this review.

The progress in this field in the last 10 years has favored the investment of the companies working in the prosthetic field to produce and commercialize myoelectric multifingered prosthetic hands, e.g., Touch Bionics iLimb ^2^, Ottobock Michelangelo^3^ and RSL Steeper BeBionic3^4^ able to provide different grip patterns by means of EMG control. Notwithstanding they are still characterized by a limited numbers of active degrees of freedom and do not provide sensory feedback to the user. They only use a position loop to control hand grasping.

**Figure 1:**
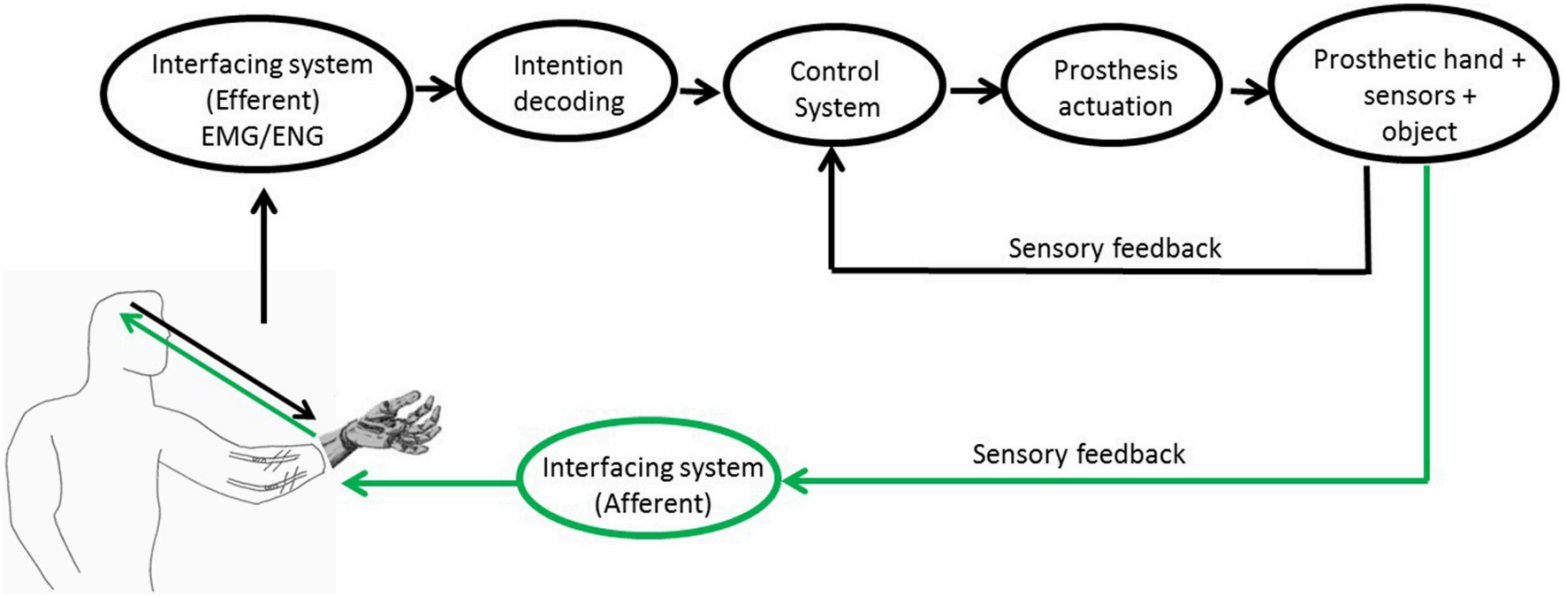
**Block scheme of the PNS-based control of a prosthetic system**. Readapted from^5^ and (Raspopovic et al., 2014).

A closed-loop control around the user (Antfolk et al., 2013b) is characterized by a *bidirectional communication* between the user and the prosthetic system (Figure 1).

The control of the prosthesis benefits from information coming from the efferent pathway, translating neural or muscular commands. In a PNS-based control of a prosthetic hand the subject intention of movement can be extracted from muscular or neural signals through EMG and ENG interfaces, respectively. Muscular commands can be acquired in invasive or non-invasive ways using epymisial or superficial electrodes, respectively. Instead, the extraction of neural signal always requires the use of an invasive technique to implant neural interfaces around (cuff electrodes) or inside the nerve (intraneural electrodes).

On the other hand, the afferent pathway is used for returning to the user the information on the interaction between prosthesis and environment (tactile perception, proprioception, pain, and temperature) (Farina and Aszmann, 2014). In Childress (1980), three different afferent pathways were proposed, based on: (i) visual or auditory feedback signals; (ii) somatic sensory signals, i.e., tactile, proprioception, vibration; (iii) feedback signals intrinsic to the prosthesis control system, which use information of the sensors embedded in the prosthesis for automatically adjusting the grasping force.

Somatic sensory signals can be generated through non-invasive or invasive interfacing techniques (Antfolk et al., 2013b; Schofield et al., 2014), such as: vibrotactile, electrotactile, mechanotactile, targeted sensory reinnervation (TSR) and neural stimulation.

Vibrotactile and electrotactile sensory substitutions (Kaczmarek et al., 1991) stimulate the skin with mechanical vibrations or local electrical currents. The reduced power consumption and the fast response to the stimulus are the main advantages of the electrotactile technique with respect to the vibrotactile one. Mechanotactile feedback consists of providing a pressure or a force normal to the skin by means of a pusher. In Antfolk et al. (2013a), it has been suggested that the amputees' ability to detect pressure stimulation exceeds their ability to discriminate vibrotactile stimulation, thus supporting the choice of mechanotactile stimulation to vibrotactile one. Furthermore, mechanical feedback is more accepted than electrotactile feedback by myolelectric prostheses users since in the electrotactile modality it is difficult to isolate and elicit a specific sensation for a specific task.

In the TSR, the skin near or over a targeted muscle (a big muscle spared by the lesion, often the *pectoralis major*) is reinnervated with afferent fibers of the remaining hand nerves: when the skin is touched, it provides the amputee with a sense of the missing arm or hand (Kuiken et al., 2004; Kim and Colgate, 2012), or else a hand map (Hebert et al., 2014a,b). The feedback is returned in a physiologically correct manner by means of tactors positioned on the residual limb (Hebert et al., 2014b). Although promising, this technique is younger than the previously described feedback methods and more invasive, thus being limited to few cases of very proximal amputation.

An alternative to the aforementioned techniques is the sensory feedback directly provided on the afferent pathway through neural electrodes, thus exploiting the natural pathways of communication between the hand and the peripheral nervous system (PNS). For this strategy, the great challenge is to recover the bidirectional communication with the PNS in amputees through the surgical implantation of neural electrodes in the upper-limb peripheral nerves. Studies on long-term amputees indicated that central pathways associated with amputated peripheral nerves retain at least some sensory and motor function (Dhillon et al., 2005). This supports the possibility of performing a natural control of the prosthesis and of returning a natural sensory feedback to the amputee in a closed loop control by means of implantable peripheral interfaces.

The paper is structured as follows: a literature review on prosthetic hand control, sensorization and neural implants for sensory feedback restoration is presented in Section II. Section III discusses the main achievements and limitations of the analyzed works and proposes a set of the main suggestions that a PNS-based prosthetic hand has to satisfy in order to recover the bidirectional communication with the PNS. Finally, Section IV draws the conclusions.

## Prosthetic hand control

### Myoelectric control

One of the first solutions of myoelectric control is the on-off control: when the EMG signals exceed a threshold, a certain prosthesis function is activated (Scott and Parker, 1988). This simple and intuitive control modality requires many sites to extract the EMG signal, one for each function to control. This condition is often prohibitive in proximal amputees and hugely restricts the number of functions to select. One way to overcome this drawback is to have different activation thresholds, but this solution (called Double-Command Control) affects the simplicity of use (Battyeet et al., 1955) and limits its application. As an alternative, the Agonist/Antagonist Control can be used. It consists of using a couple of electrodes on agonist-antagonist muscle pairs (Popov, 1965). The contraction of one muscle is associated to the motion of opening with constant speed, while the contraction of the other muscle controls the closure; simultaneous muscle pair contraction (co-contraction) allows switching from one function to another. Setting an intermediate threshold of the muscle contraction it is possible to obtain a two-speed motion (i.e., Two-Speed Control) of the prosthesis (slow and fast) to accommodate fine movements.

Proportional control (Fougner et al., 2012) permits to vary force and speed proportionally to the amplitude of the recorded EMG signal. Hence, the voltage command for the motors is taken as proportional to the contraction intensity. Measuring EMG signals from agonist/antagonist muscles is a common procedure in proportional control and co-contraction of the muscle pair is used to select the degree of freedom to control.

The main limitation of agonist/antagonist control consists of the limited number of independently controllable DoFs (far from the multifunctional control of the human hand). Anyway, thanks to its simplicity and robustness, it results to be the most adopted control option for myoelectric prostheses in commercially available systems as well as in clinical applications (Jiang and Farina, 2014).

Two additional techniques have been developed in the framework of myoelectric control, i.e. the Targeted Muscle Reinnervation (TMR) (Hijjawi et al., 2006) and the recent nerve transfer in brachial plexus injury (Aszmann et al., 2015). In the TMR Hijjawi et al. (2006), the remaining arm nerves are reallocated to residual chest or upper-arm muscles that are no longer biomechanically functional due to the amputation. Once re-innervated, these muscles serve as biological amplifiers of motor commands from the transferred arm nerves and provide physiologically appropriate EMG signals for the arm control. This procedure is especially applied to subjects with very proximal amputation, which usually control the motors of the prosthetic arm through switches actuated with residual shoulder movement or myoelectric signals acquired from muscles of the chest and back. With respect to these control techniques, TMR presents several advantages, such as improvements in function (measured both objectively and subjectively), ease of use, simultaneous control of more than one DoF, fast and seamless motion (Miller et al., 2008).

In Aszmann et al. (2015) nerve transfer in brachial plexus injury is presented. It is defined “bionic reconstruction.” After free functioning muscle transfer procedure^6^ for the restoration of shoulder and elbow functions, the hand muscle activity has been restored selectively transferring the nerves in order to optimize the number of electromyographic sites. The surgical procedure and the rehabilitation program allowed the improvement of EMG activity and the maximization of the prosthetic hand functions.

Over the years, several studies for improving myoelectric control performance have been carried out. The development of pattern recognition techniques aimed at increasing the number of controllable DoFs (and consequently the number of feasible functions) keeping low the number of utilized electrodes (Roche et al., 2014). Myoelectric control based on pattern recognition techniques resorts to supervised machine learning algorithms (Zecca et al., 2002): in an initial training phase the system learns to associate different hand gestures to different myoelectric patterns based on the phantom limb effect. This association is then adopted in the daily use of the prosthesis.

The first step of pattern recognition consists of the feature extraction: the main components of the recorded myoelectric signal are identified and selected in a time window between 150 and 250 ms, depending on the skill of the subject (Smith et al., 2010) The main purpose of this step is to enhance the information content, retaining information about contraction discrimination while discarding the irrelevant ones,. Feature extraction techniques are typically in the time domain and in the frequency domain (Cloutier and Yang, 2013).

Commonly used time domain techniques are: mean absolute value (MAV) (Hudgins et al., 1993; Zardoshti-Kermani et al., 1995; Park and Lee, 1998), zero crossing (ZC) (Hudgins et al., 1993), waveform length (WL) (Farry et al., 1996), root mean square (RMS) (Hudgins et al., 1993) and slope sign change (SSC) (Farry et al., 1996), AR model (Graupe and Cline, 1975).

Techniques in the frequency domain are more accurate but also computationally more demanding than time-domain techniques. They include: Short-Time Fourier Transform (STFT), wavelet transform (WT) (Vetterli and Kovacevic, 1995; Akay, 1997; Englehart, 1998; Pattichis and Pattichis, 1999; Karlsson et al., 2000; Sparto et al., 2000) and wavelet packet transform (WPT) (Coifman and Wickerhauser, 1992).

In order to reduce the computational complexity and, at the same time, increase the performance of the subsequent classification (Hargrove et al., 2009), the dimensionality reduction through the Principal Component Analysis (PCA) can be applied to EMG signals (Chan and Green, 2007). Classification follows feature extraction and dimensionality reduction. It is responsible for the decoding of the patient motor intention.

Pattern recognition classifiers (Bishop, 2006; Kotsiantis et al., 2007; Cloutier and Yang, 2013) can be grouped in the following main categories, sorted by increasing complexity: linear classifiers, such as Linear Discriminant Analysis (LDA) or Perceptron or Support Vector Machine (SVM), non-linear classifiers, such as Non Linear Logistic Regression or SVM with non-linear kernels, and Multilayer Perceptron or Multilayer SVM.

The main difference between linear and non-linear classifiers is the shape of the decision boundaries that divide the features space in classes: straight line (or plane or hyper-plane) in the linear case and curved in the non-linear one.

An extensive inspection of classifiers can be found in Scheme and Englehart (2011) and Zecca et al. (2002). In the literature a large number of feature sets and classification algorithms employed in myoelectric control have been investigated and compared in detail. Notwithstanding, there is no clear evidence of the superiority of one classifier over the other ones; it is shown that classifiers can reach similar performance in terms of offline accuracy, provided that an appropriate feature set and an adequate number of sampling sites of the EMG signal are used (Scheme and Englehart, 2011). In Hargrove et al. (2007) the effects of the choice of feature sets over classifier performance are in-depth investigated. Moreover, methods based on “raw” filtered EMG signals have been recently proposed; they allow considerably decreasing the time for feature extraction and skipping the feature reduction step without significant loss of system performance (Nazarpour, 2005; Dohnalek et al., 2013).

However, it is worth observing that the viability of pattern recognition in a clinical setting should consider that off-line accuracy could not correspond to real-time performance (Lock et al., 2005). The analysis of real-time performance of pattern-recognition algorithms is required, as proposed in Kuiken et al. (2009); in it a pattern recognition algorithm based on a LDA classifier was used to move a virtual prosthesis and performance was measured through the time taken to select and complete the desired motion, and the percentage of motion successes. The classification process can include a learning phase (through unsupervised machine learning algorithms). This allows the system to improve the on-line classification performance and robustness but it increases system complexity.

In addition, in laboratory trials, during experimental tests on healthy subjects, pattern recognition algorithms can reach high percentage of success, up to 95%, with more than 10 classes used (Scheme and Englehart, 2011) and offline data analysis. On the other hand, the continuous use of the prostheses requires facing situations that are different from ideal laboratory conditions (Jiang and Farina, 2014), e.g., there are changing in the arm posture, electrodes position and fatigue. All these factors lead to a reduction of the performance of these techniques. Other disadvantages to consider are very long learning sessions and a different psychological attitude of the amputee when the prosthesis is used in the real context.

Despite the first proposed control scheme based on pattern recognition dates back to the late sixties (Finley and Wirta, 1967; Lyman et al., 1976), only recently its clinical viability appears to be closer (Scheme and Englehart, 2011), especially thanks to the improvements achieved in signal processing, multichannel instrumentation and microprocessor technology. Indeed, the first prostheses control device based on pattern recognition and surface electrodes (COAPT^7^) is commercially available since January 2015. Developed in cooperation with Dr. Todd Kuiken and NECAL laboratory at the Rehab Institute of Chicago, it is undergoing clinical trials in several US rehabilitation centers, also in conjunction with TMR. Pattern recognition offers the notable advantage of enabling the simultaneous, independent control of multiple DoFs. Since 1973 (Herbert et al., 1973), various approaches have been attempted in this direction. A parallel set of LDA classifiers has been proposed in Young et al. (2013) to provide classification of simultaneous wrist and hands movements. The proposed strategy demonstrated superior classification performance respect to three methods (i.e., single LDA classifier, three LDA parallel classifiers, SVM parallel classifiers) previously proposed in literature for applying pattern recognition classification to combined motions (Davidge, 1999; Baker et al., 2010; Boschmann et al., 2011). In the recent work (Ortiz-Catalan et al., 2014b) it has been shown that every classifier can be potentially employed in the control of multi- DoFs if properly arranged in a distributed topology. Also, a control method based on the so-called Non-negative Matrix Factorization (NMF) has been proposed, which permits the simultaneous and proportional control of 2 degrees of freedom (in particular flexion/extension and prono/supination of the wrist). It requires only an initial calibration and has a success rate of around 95% (Jiang et al., 2014). Despite the encouraging results, the main limitation of this approach is the high number of EMG electrodes (i.e., 16 electrodes).

The use of pattern recognition-based controllers has been clinically limited for a number of reasons, e.g., the complex nature of forearm muscles synergies, the inherent cross talk in the surface signal, and the displacement of the muscles during contraction. In order to overcome these problems, the substitution of surface EMG with intramuscular EMG has been proposed. Implantable Myoelectric Sensors (IMES) have recently been implanted in a transradial amputee (Pasquina et al., 2015) to provide intuitive and stable myoelectric control. Preliminary results have demonstrated the possibility to record voluntary individual muscle activity and wirelessly transmit this information to control 3 DoFs of a prosthetic device (i.e., wrist pronation/supination, thumb adduction/abduction and finger flexion/extension). However, IMES have to be used with caution, as they cannot be employed when the sensing sites are very close each other or else the target muscle is small or thin.

### Hand sensorization

The sensors usually embedded in artificial hands, for prosthetics as well as robotic applications, typically belong to two different categories: position sensors, for providing hand proprioceptive-like information, and force/tactile sensors, for measuring the interaction with the external environment.

Over the years, a number of researchers have developed tactile sensors based on a huge variety of functioning principles. Force measurements as well as surface properties (e.g., hardness, roughness, temperature etc.) can be inferred from tactile sensors.

**Figure 2:**
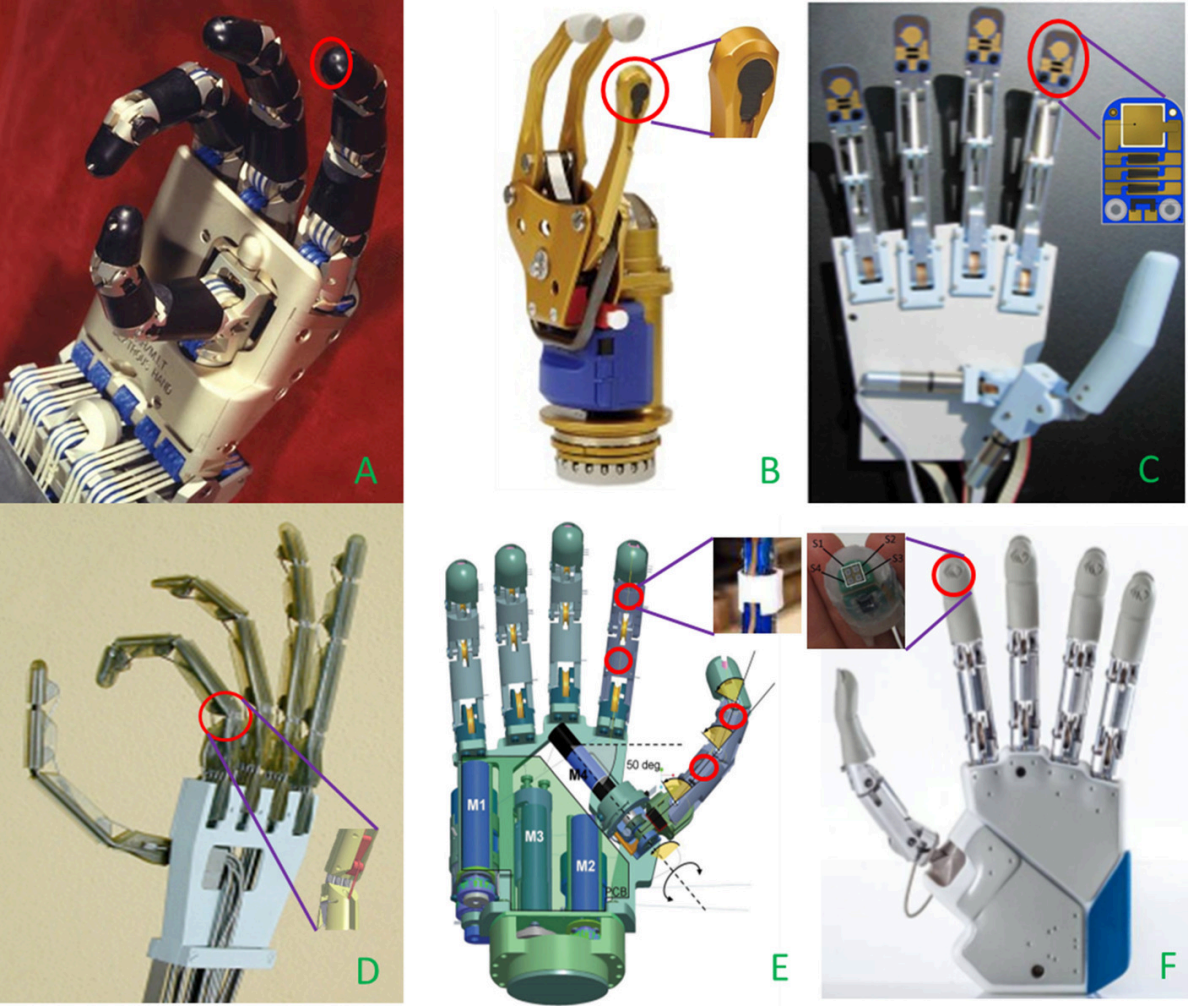
**Details of the tactile sensors (red circles) embedded in prosthetic hands. (A)** Piezo-resistive sensor on fingertips of UTAH-MIT Hand^8^ (Courtesy of Compuer Histiry Museum) **(B**) Stain gauges based SUVA system integrated in the thumb of SensorHand Speed^9^ **(C**) Tactile sensor with piezo-electric, piezo-resistive and thermal sensing units mounted on Southampton Hand (Cotton et al., 2007); **(D)** Strain gauge placed on PIP joint of UB Hand III (Lotti et al., 2004); **(E)** Optical sensors on thumb and index of SmartHand (Cipriani et al., 2011); **(F)** MEMS tactile sensors on fingertips of IH2 Azzurra^10^ (Courtesy of Prensilia srl).

Providing a prosthetic device with reliable tactile information still represents a very tough challenge in the robotic and prosthetic fields. In the following, a brief overview of transducing principles applied to tactile sensing is illustrated, together with prosthetic hands employing one or more of such principles (see Table 1). Piezoresistivity is one of the more commonly adopted principles for tactile sensors being quite simple and cheap. Piezoresistive force sensors were employed in the fingertips of the Utah-MIT Hand (1986) (Figure 2A), one of the first developed robotic hand for prosthetic use. The sensing element (Allen et al., 1990) comprised 256 elements organized in a 16 × 16 matrix inserted between two deformable Kapton sheets. Similarly, the Belgrade-USC Hand (1988), used piezo-resistive elements located in the fingertips for force measurements and contact detection (Martell and Gini, 2007).

**Table 1:** **Prosthetic hands proposed in the literature and their features**.

**Hand name**	**Achievable grasps**	**DoA**	**Sensorization**		**Actuation method**	**Weight [g]**	**Joint Coupling method**	**Grip force [N]**		
			**Force/Slippage**	**Position**				**Power**	**Precision**	**Lateral**
SensorHand by Ottobock	Power	1	SUVA Sensor System^**^	–	DC Motor^*^	350–500	Fixed Pinch^*^	100	NA	NA
i-Limb by Touch Bionics	Power, Precision, Lateral, Hook, Finger-point	5	–	Encoders^**^	DC Motor—Worm Gear^*^	443–515	Tendon linking MCP to PIP^*^	100–136	-	21–35
Bebionic by RSL Steeper	Power, Precision, Lateral, hook, finger-point	5	–	Encoders^**^	DC Motor – Lead Screw^*^	550–598	Linkage spanning MCP to PIP^*^	140.1	36.6	26.5
Michelangelo by Ottobock	Opposition, Lateral, Neutral Mode	2	–	–	DC Motor—Central Drive Actuation System^*^	420	Cam design with links to all fingers^*^	70	70	60
IH2 Azzurra by Prensilia	Power, Precision, Lateral, Hook, Finger-point	5	–	5 encoders 5 current sensors 10 limit switch Hall sensors	DC Motor—Lead Screw	640	Tendon driven mechanism	35	-	7
Utah/MIT Hand	Power, Precision, Lateral, Hook, Finger-point	32	Tendon tension sensor Piezo-resistive force sensors	Hall-effect joint position sensors	Pneumatic Actuators	?	Tendon driven mechanism	Tip forces about 31		
Belgrade/USC Hand	Power, Precision, Lateral	4	Piezo-resistive force sensors	Potentiome-ters	DC Motors	–	Linkage driven mechanism	–		
NTU Hand	Power, Precision, Lateral, Hook, Finger-point	17	–	Joint position sensors	DC Micromotor	1569	Gear Trains mechanism	–		
Gifu Hand III	Power, Precision, Lateral, Hook, Finger-point	16	Tactile sensors (859 detecting point)	Magnetic encoders	DC Motors	1400	Gear Train and linkage mechanism	Thumb fingertip force: 3.7 Other fingertips force: 3.4		
Southampton Hand	Power, Precision, Lateral, Hook	6	FSR sensors Piezoelectric slip sensors Thermistors	Encoders	DC Motors	400	Worm wheels gears	Fingertips forces: 9		
UB Hand III	Power, Precision, Lateral, Hook, Finger-point	20	Tendon force sensors Load cells	Potentiometers	DC Motors	–	Tendon driven mechanism	Tendon force: 70		

*NA, not applicable, – information not available*,

* Information extracted from Belter et al. (2013);

** *Information extracted from Balasubramanian and Santos (2014)*.

In the framework of the TIDE project the MARCUS (1992) hand, a device with a three-fingered polyarticulated for myoelectric prosthesis, was developed (1992). The hand was equipped with position, force and slip sensors while a sensor-based control allows it to maintain a stable grasping of the object without affecting the user attention (Davalli and Sacchetti, 1993).

Force Sensitive Resistors (FSR) technology exploits piezoresistivity too. They have been largely used in prosthetics since 90s. FSR sensors are made of polymeric thin films with variable electrical resistance, their small size are ideal for hand prosthetic usage. They suffer from a certain non-linearity in the response; however, their great sensitivity at low forces constitutes a remarkable feature from a tactile point of view (Chappell, 2011). In the Gifu Hand II, 2001 (Figure 2B) a total number of 624 active elements collecting the tactile signals thanks to a layer of conductive ink whose electrical resistance changes in relation to the applied stress on the active elements surface. In addition, 6-axis force sensors have been placed on each fingertip (Kawasaki et al., 2002). In the successive release of the hand, the Gifu Hand III (Mouri et al., 2002) 235 additional active elements have been used.

In the first years of this century, embedded solutions for force/tactile sensors have been progressively adopted, with particular attention to the problem of slippage phenomena occurring during manipulation and grasp tasks. The piezoelectric materials are particularly appropriate for slippage detection, because of the high sensitivity to micro-vibrations and the low cost (Cotton et al., 2007). However, their main drawback is that vibrations due to other events, different from slippage, might lead to inappropriate activations of the sensors, and as a consequence, to noise generation. Moreover, their output is temperature dependent, particularly evident in sensors based on polymeric materials [e.g., polyvinylidene fluoride (PVDF)], that are widely exploited as tactile sensors.

Southampton REMEDI Hand, 2001 (Figure 2C), relies on two different physical principles in order to gain tactile information from the interaction with the objects (Cotton et al., 2007): FSR sensors, employed for force estimation, and a piezoelectric layer (lead zircontetitanate, PZT) on the fingertips to detect slippage.

Alternatively, traditional force sensors based on strain gauges can be utilized. Load cells are able to provide accurate force measures: the UBH III hand, 2003 (Figure 2D) has been mounted with some load cells located close to each actuated phalanx (Lotti et al., 2004).

Lately, the research interest in optoelectronic technology is growing. It offers a number of advantages, such as optimal accuracy, good linearity, absence of electromagnetic interference and hysteresis in sensors measure. Typical disadvantages derive from a more complex electronics, from the typically high currents needed for supplying photodiodes, and from the light attenuation deriving from microbending in the walls of the light guide. The idea to use an optical sensor array to detect slipping was implemented in a commercial hand in 1998 at INAIL prostheses center, but the impact on the cosmetic glove was unacceptable (Davalli et al., 1998). The Smart Hand, 2008 (Figure 2E), is a good example of application of optical sensors array. It embedded 4 optoelectronic sensors, 2 in the proximal and intermediate phalanges of the index, and 2 in proximity of the metacarpophalangeal (MCP) and interphalangeal (IP) joints of the thumb (Persichetti et al., 2007; Cipriani et al., 2011). The structure of the sensor included an infra-red (IR) photodiode, a phototransistor and a silicone cover.

Finally, tactile sensors can be fabricated by means of MEMS technique, with considerable reduction of sensors bulkiness. This is the case of the new generation of IH2 Azzurra (Figure 2F), developed by Prensilia s.r.l. It can be provided with a 2 × 2 matrix of MEMS piezo-resistive sensors embedded in each fingertip: each sensitive element of the matrix included 4 active units, for a total of 16 units in a surface of 0.22 cm^2^, corresponding to 72 units/cm^2^ [which close to the density of Merkel Disks in the human fingertip (70 units/cm^2^)]. Discrimination of ridged surfaces has been performed by means of such sensors (Oddo et al., 2011), thus pointing out their elevate potential as slip sensors.

Other transducing principles have been investigated by researchers, e.g., capacitive, magnetic, quantum tunnel, thermic, acoustic tactile sensors, and fiber optic based sensors (Dahiya et al., 2009; Francomano et al., 2013; Saccomandi et al., 2014). Although considered promising technologies, there are available prosthesis integrating similar tactile sensors, yet.

On the other side, looking at the panorama of commercially available prosthetic hands, only the Sensorhand Speed (2002) by Ottobock^11^ (Figure 2E) is provided with a slippage detecting system in the thumb, namely the SUVA Sensor System. It is made of three sensors disposed at angles of 120° and is able to detect slippage by constantly monitoring the position of the gravity center of the object and, consequently adjusting the grasp force level in case of necessity.

More recent models of multifingered commercial hands (e.g. the i-Limb by Touch Bionics^2^ (2007), the Bebionic by RSL Steeper^3^ (2010) and the Michelangelo by Ottobock (2010)^4^ are not provided either with force or tactile feedback. Different grasps can be executed thanks to predefined grip patterns and hand intrinsic compliance.

Moreover, the sensors selection must to consider the presence of the cosmetic glove and the characteristics of the glove itself, the contact area and the capability to accept direct load.

### Neural control and sensory feedback restoration

Neural control of prosthetic hands can be achieved by recording electrical activity of the hand (Navarro et al., 2005). ENG-based control is grounded on the evidence that activity related to movement is present in M1 and S1 cortical areas still after the amputation (Reilly et al., 2006). The pioneering work in Dhillon et al. (2004) showed that volitional motor nerve activity can be recorded by means of intrafascicular electrodes implanted in the amputee's stump. Over the years, several studies were performed in order to understand the possibility of restoring sensory feedback in individuals with limb loss by means peripheral nerve stimulation. One of the first experiments has been performed in Clippinger et al. (1974), where an induction-powered radio receiver-pulse generator was implanted for motor stimulation of the median nerve of 15 patients. Their experience provided support to the possibility to restoring the sensation of the pressure applied to the grasped object. Subsequently, several studies attempted to elicit sensations by means of neural electrodes (i.e., cuff, Polasek et al., 2009, or intraneural Ochoa and Torebjork, 1983; Dhillon et al., 2004).

In Dhillon and Horch (2005) for the first time a bidirectional control of a prosthetic hand was developed through the use of intraneural electrodes. Although they demonstrated the possibility to use implanted peripheral nerve electrodes (i) to produce discrete touch and movement sensations and (ii) to record motor neuron activity usable as hand control signals, this study did not explore closed-loop, non-visual control of the prosthesis. More recently, experimental studies were performed to demonstrate the feasibility of restoring the natural tactile sensory feedback by means of neural stimulation in upper limb amputees using hand prostheses (Rossini et al., 2010; Ortiz-Catalan et al., 2014a; Raspopovic et al., 2014; Tan et al., 2014). They are described in the following and summarized in Table 2.

**Table 2:** **Summary of results on neural implant studies for sensory feedback restoration**.

	**Rossini et al., 2010**	**Raspopovic et al., 2014**	**Ortiz-Catalan et al., 2014a**	**Tan et al., 2014**
Number of subjects	1	1	1	2
Experimental period	4 weeks	4 weeks	up to 16 months	up to 24 months
Electrodes	tf-LIFEs (thin-film Longitudinally-implanted Intra Fascicular Electrodes)	TIMEs (transversal intrafascicular multichannel electrodes)	Cuff electrode (Ardiem Medical)	FINE (flat interface nerve electrodes) Cuff electrode (Ardiem Medical)
Number of electrodes	4	4	1	Subject 1: 2 FINEs, 1 cuff Subject 2: 2 FINEs
Nerves	Median and ulnar nerves	Median and ulnar nerves	Ulnar nerve	Subject 1: medial and ulnar nerves Subject 2: medial and radial nerves
Trains of pulses	Rectangular cathodal pulses	Rectangular cathodal pulses	Single active charge-balanced biphasic pulse	Square electrical pulses
Frequency	10–100 Hz	50 Hz	8–20 Hz	10–125 Hz
Current	10–100 μA	maximum stimulation current: 240 μA (at 100 μs) for the index finger and 160 μA(at50 μs) for the little finger.	30–50 μA	1.1–2 mA
Pulse width	10–300 μs	–	–	24–60 μs
Charge	0.1–4 nC	Median nerve: 14-24 nC Ulnar nerve: 4-8 nC	100-180 μA	Subject 1: 40.7–95.5 nC Subject 2: 95–141 nC
Elicited hand areas	Figure 3A	Figure 3B	Figure 3C	Figures 3D–E
Grasping task	Power grip, pinch grip, little finger flexion	Palmar grasp, pinch grasp, ulnar grasp	Tripod grasp during arm oscillation, power grasp in different limb position	

The work in Rossini et al. (2010) showed the feasibility of (i) controlling a hand prosthesis by means of the neural signal directly extracted from median and ulnar nerves of an amputee; (ii) using afferent neural stimulation to elicit tactile sensory feedback.

In Raspopovic et al. (2014) the bidirectional control of a prosthetic hand was developed and used to recognize object features and positions during grasp. The control consisted of: (i) decoding the user intentions through an EMG interface and (ii) eliciting tactile sensory feedback activated by the sensors on the hand through a neural interface. A proof-of-concept of the closed loop control with the return of a sensory feedback was given by this experimental trial also recording an improvement of the prosthesis control performance.

The work in Ortiz-Catalan et al. (2014a) demonstrated the improvement of hand controllability using implanted EMG sensors and the feasibility of eliciting tactile sensations in chronic implants. The electrodes were permanently implanted in the subjects with results reported up to 16 months after surgery. Finally, the work in Tan et al. (2014) proved the feasibility of eliciting tactile sensations in a stable manner up to 24 months and demonstrated that the perceived sensations and the perceived areas can be modulated changing pulses parameters. This study did not evaluate the aspects related to the prosthesis control.

#### Study 1: Experimental study on neural control of hand prosthesis (Rossini et al., 2010)

The human experimentation in Rossini et al. (2010) has been carried out in 2008 on a 26 years old amputee and had the aim to test a prosthetic hand directly controlled by neural signal by means of thin-film intraneural electrodes (thin-film Longitudinally-implanted Intra Fascicular Electrodes—tf-LIFE4s). During 4 weeks of experimentation the subject has been able to send motor commands to the prosthetic hand in order to directly control it and receive sensory information from the sensors in the hand by means of the neural electrical stimulation (only for the first 10 days).

Four tf-LIFE4s (Hoffmann and Kock, 2005) electrodes were implanted parallel to the main axis of the nerve through a surgical intervention: 2 in median nerve and 2 in ulnar nerve.

The prosthetic hand used during the experiment was a prototype of the CyberHand (Carrozza et al., 2004). It was endowed with position and tension sensors to measure finger positions and cable tensions, respectively, during finger flexion/extension.

##### Experimental protocol

The experimental protocol consists of two main phases: (a) recording of motor output from efferent fibers in order to control the prosthetic hand and (b) sensory stimulation of afferent fibers in order to elicit sensation.

The delivering of electrical current in the tf-LIFE4s electrodes allowed to elicit sensations. Details regarding stimulation parameters are shown in Table 2. The 32 electrodes contacts have been mapped in order to identify the elicited sensations on the afferent fibers (Rossini et al., 2010). The stimulation was not driven by sensors embedded in the prosthesis, but was artificially triggered by the experimenters.

The perceived sensation have been quantified by the amputee with a number between 1 (minimal sensation perceived) and 5 (discomfort).

##### Results

The discrimination of the recorded neural signals was more successful in case of recordings from multiple contacts in both nerves. The correct classification of each movement was related to a learning effect, with an improvement from 75 to 85% in 2 days.

**Figure 3:**
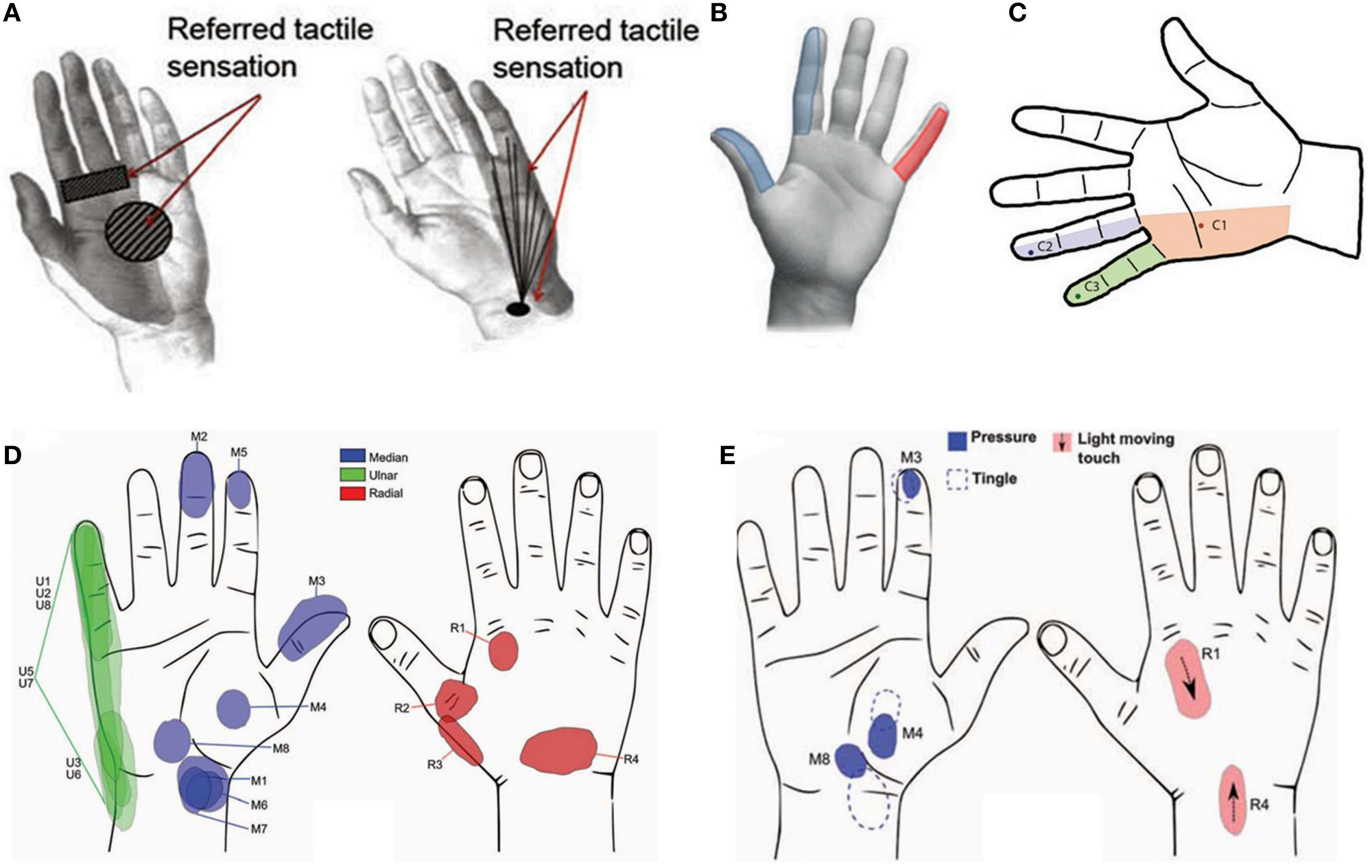
**(A)** Perceived localization of sensation after—median nerve tf-LIFE4 stimulation on left and after ulnar nerve tf-LIFE4 stimulation on right (Rossini et al., 2010); **(B)** Elicited hand areas in Raspopovic et al. (2014); **(C)** Tactile perception via neurostimulation (Ortiz-Catalan et al., 2014a). The dark points represent the electrode-specific projected field repeatedly reported (over 11 months) for a single pulse at stimulation threshold; **(D)** Sensation locations after threshold stimulation at week 3 post-op (Tan et al., 2014). The letter represents the nerve and the number represents the stimulus channel; **(E)** Pressure tactile perception on varying of impulse duration (Tan et al., 2014).

Different electrode contacts allowed eliciting different tactile sensations (Figure 3A) if an electrical current, under the safety limits, was applied. The modulation of stimulus frequency permitted to modulate the intensity of sensation on a logarithmic scale, as also demonstrated in Dhillon et al. (2004). The minimal charge to produce the perceived sensation increased during the days probably in relation to the fibrotic reaction caused by the electrodes (Rossini et al., 2010).

The experimental training also allowed improving the symptoms of the phantom limb pain evaluated by means of McGill Pain Questionnaire (sfMcGill), Present Pain Intensity (PPI) e Visual Analog Scale (VAS).

#### Study 2: Experimental study on bidirectional control of hand prostheses (Raspopovic et al., 2014)

In Raspopovic et al. (2014) the results of a surgical implant of transversal intrafascicular multichannel electrodes (TIMEs, Boretius et al., 2010) for the bidirectional control of the hand prosthesis are reported. The experimental protocol had the two-fold aim of restoring the sense of touch in a volunteer subject with transradial amputation by means of the TIME electrodes, and exploiting the tactile information to close the control loop of the artificial hand on the amputee.

The bidirectional control of the prosthesis relied on: (i) a myoelectric control of the prosthesis on the efferent pathway and (ii) a sensory loop that, reading the hand sensor readouts, was able to elicit tactile sensations on the afferent pathway.

Four TIME electrodes (Boretius et al., 2010) have been implanted in median (2 electrodes) and ulnar (2 electrodes) nerves in order to almost completely cover the sensorial areas of palm and hand.

The Prensilia IH2 Azzurra hand (Figure 2F) provided with tension sensors on the tendons has been used during the experiments. The tension measure of the finger cable provided an indirect measure of the force exerted by the index finger during the interaction with the objects.

##### Experimental protocol

Two main phases can be distinguished in the experimental protocol. During the first phase the peripheral nerves have been stimulated in order to identify all the possible elicitable sensations and the related territories where the sensation was referred. In the second phase user functioning of the closed loop control was verified; thus, intention were decoded and grasping tasks were performed.

The tactile sensory feedback has been evaluated during 4 weeks so as to steadily monitor the elicited sensation during the electrical stimulation. The range of charge capable to produce sensation on ulnar nerve remained stable for the whole experimental period while it increased for the median nerve (Raspopovic et al., 2014).

Table 2 specifies the details regarding stimulation parameters.

Validation tests of recognition of object position and properties have been carried out. The amputee (blindfolded and acoustically isolated) has also been subjected to trials of fine real-time force control. To this purpose, he has been asked to apply three different force levels to a chamber provided with pressure sensor and use the sensorial feedback to modulate the force during pinch, palmar and ulnar grasps (Raspopovic et al., 2014).

##### Results

The hand areas elicited from the electrical stimulation of medial and ulnar nerves are shown in Figure 3B.

The amputee has been able to voluntarily control three levels of pressure exerted on the object with index and little fingers with a success rate >90%.

The performance of the prosthetic hand has been compared with the healthy hand during the same task with visual and acoustic feedback and without neural stimulation. The amputee performing task with the prosthesis and tactile feedback (without visual and acoustic feedback) reached performance closer to the ones achieved performing the same tasks with the healthy hand than to the ones achieved performing the same tasks with the prosthesis with only visual and acoustic feedback, but without tactile stimulation (Raspopovic et al., 2014).

The amputee has been asked to grasp a spherical object with cylindrical, ulnar and median grasp without acoustic or visual feedback. The subject has been able to recognize the position of an object placed on the palm and to define the most appropriate grasp to exert on it. The mean accuracy observed over 52 different trials has been 97% (Raspopovic et al., 2014).

The experimental trials on the recognition of object properties by exploiting the sensory feedback required recognizing object form and consistence. The amputee has been able to recognize the consistence of three different objects with performance of 78.7% (Raspopovic et al., 2014). Moreover he identified three object shapes with mean accuracy of 88%.

#### Study 3: Experimental study on long-term sensory feedback and motor control of hand prosthesis (Ortiz-Catalan et al., 2014a)

One patient with transhumeral amputation has been treated with an osteointegration procedure in January 2013. One cuff neural electrode by Ardiem Medical has been implanted on the ulnar nerve in order to restore the sensory feedback. Moreover two bipolar epimysial EMG electrodes (biceps and triceps muscle) and four monopolar epimysial EMG electrodes (biceps, triceps, brachialis muscle) have been implanted with the aim to collect myoelectric signals for controlling a prosthetic hand (Figure 3C; Ortiz-Catalan et al., 2014a). The positions of EMG and cuff electrodes have been defined in order to maximize the contribute of active sites and the signal-to-noise ratio (Navarro et al., 2005; Ortiz-Catalan et al., 2012).

The tests have been carried out with the patient myoelectric hand during working and daily living activities.

##### Experimental protocol

The differences in performance between superficial EMG sensors and epimysial ones have been evaluated in four different sessions meanwhile the stimulation thresholds have been evaluated seven times.

The tactile sensation has been investigated through electrical stimulation of the nerves during 11 months. The sessions of stimulation have been carried out two times in a day at 5, 7, 8, 10, 12, 14, and 16 months after implantation (Ortiz-Catalan et al., 2014a).

McGrill Pain Questionnaire has been employed to evaluate the evolution of the phantom limb pain and the use of the prosthesis. It has been administered 2 months before the implant and 10–16 months after the implant.

##### Results

Eight different movements (hand opening/closing, wrist pronation/supination, wrist and elbow flexion/extension) have been performed with an accuracy of 94.3% (σ = 1.6%).

The results achieved with the prosthetic hand were comparable with those obtained with the healthy hand. High controllability has been demonstrated with a rate of completion of 100% and a mean efficiency of the path of 90.5% in 3.8 s. In case of test involving hand and wrist the efficiency has been of 96.3% in 3.0 s.

As regards the sensory feedback the experiments showed that the tactile sensation elicited with electrical stimulation has been unchanged all along the 11 months of experiments. A superficial tapping has been perceived by the amputee in some areas of the hand (Figure 3C) during electrical stimulation with current pulses between 8 and 10 Hz. Pulses above 20 Hz elicited tingling sensations. The involved areas have been expanded increasing the current of the single pulse from 30 to 50 μA or employing a pulse train with frequency above 20 Hz. The intensity of the stimulus increased with the growing of the frequency from 7 to 10 Hz.

Compared to the use of superficial EMG sensors, the controllability of the prosthesis has been improved and the time of usage of the robotic hand has been increased of 6 h per day. Moreover the phantom limb pain decreased of 40%.

#### Study 4: Experimental study on long-term neural touch perception and task performance improvement (Tan et al., 2014; Schiefer et al., 2015)

Two experimental studies have been carried out from the same group with the same patients (Tan et al., 2014; Schiefer et al., 2015). In the former two amputee subjects have been involved in order to verify the possibility to elicit tactile sensory feedback for long period, up to 24 months. Moreover the effect of sensory feedback on task performance has been assessed in the latter (Schiefer et al., 2015).

The first patient (male of 49-year-old) has been implanted with two FINE (flat interface nerve electrodes) (Tyler and Durand, 2003) cuffs with 8 contacts in median and ulnar nerves and one CWRU (Case Western Reserve University) spiral electrode (Naples et al., 1988) with four contacts in radial nerve, with totally 20 active sites.

Two FINE cuffs (Tyler and Durand, 2003) with 8 contacts have been implanted in median and radial nerves of a second subject (male of 46-year-old), with totally 16 active sites.

The Ottobock SensorHand Speed has been instrumented on thumb, index and middle with (i) low-profile force sensors (Tekscan FlexiForce A201) and (ii) bend sensor measuring hand aperture.

##### Experimental protocol

The stimulation experiments have been started 3 weeks after surgical implant with weekly sessions applying stimulation through each contact for up to 10 s. Different stimulation strategies have been evaluated using time invariant parameters (set to a fixed values) and time-variant pulse width.

Functional tests have been carried out using SensorHand Speed and thin FlexiForce sensors mounted on thumb and index tips during the task to pluck the stem off of a cherry.

Details regarding stimulation parameters are shown in **Table 3**. The minimum perceived threshold has been identified during stimulation with constant frequency of 20 Hz, by increasing the intensity of stimulation.

The EMG signals have been used to control the speed of hand opening and closure. Three different functional tests have been carried out to assess the impact of the sensory feedback on the prosthesis use. The first test evaluated the subject's ability to distinguish the position of a wooden block during index/thumb and middle/thumb pinch grip. A modified version of the box and blocks test has been performed to verify if the amputee was able to locate and move a block. The Southampton Hand Assessment Procedure (SHAP) has been applied with and without sensory feedback, assessing the amputee ability to perform Activities of Daily Living (Schiefer et al., 2015).

##### Results

The elicited sensations have been described by the amputees and the involved areas have been indicated on a hand picture (Figure 3D).

On subject 1 electrical stimulation on 19 active sites elicited sensations in 15 different locations on the hand. The second subject perceived sensations in 9 areas of the hand from 14 channels of the electrodes. The elicited sensations and the locations on the hand were stable and repeatable throughout the study.

A standard stimulation based on trains of rectangular waves with constant amplitude, frequency and length induced in both subjects sensation of paresthesia.

The modulation of the pulses produced sensation of pulsing pressure in the amputees (Figure 3E). A correspondence between the frequency of the pulsing pressure and the frequency of the modulation has been retrieved during the experiments, as well as between the perceived intensity of the pulse and the pulse width. The natural sensory perception has been replaced by a sensation of tingling when the pulse width increased beyond a maximum value.

The two subjects revealed different and independent tactile sensations (pulsing pressure, light moving touch or tapping for the subject 1 and pulsing pressure and vibration for subject 2) in small and defined areas of the hand, included thumb and fingertips. Moreover the sensations were coherent with the stimulated nerve.

The minimum charge necessary to elicit a tactile sensation was stable during the 8 weeks after the implant. The thresholds did not show significant change over time and no information were reported up to 68 weeks.

During the first test the subjects revealed similar performance with and without feedback (pressure and aperture) while amputees confidence increased with sensory feedback. The modified box and blocks test demonstrated that performance with sensory feedback and without vision was similar to performance with vision and without sensory feedback. Despite the SHAP score has been improved with sensory feedback, mainly during Power, Tip, and Lateral grasps, both subjects indicated a major focus on visual feedback than tactile feedback during the assessment.

Stimulation parameters are detailed in Table 2. Using LIFE (Rossini et al., 2010) or TIME (Raspopovic et al., 2014) electrodes, frequency stimulation has been between 10 and 550 Hz, while current injected has been between 10 and 240 μA. In the experimentation described in Ortiz-Catalan et al. (2014a) spiral cuff electrodes have been used, and frequency has been set between 8 and 20 Hz, while current has been varied between 80 and 180 μA. In all these cases, current injected has been less than the safety threshold of maximum current supported by the electrodes.

## Discussion

Current research effort in prosthetic field reveals the trends and the potential benefits provided by developing prosthetic devices able to restore the natural bidirectional communication between the hand and the user. This can be achieved by means of a closed-loop control on the patient that takes input from him/her through the efferent pathway and return sensory feedback to him/her on the afferent pathway.

Ideal bidirectional hand prostheses should be composed of the following main components (Figure 1): (1) the peripheral interface responsible for the bidirectional communication with the PNS; (2) the control subsystem driving the commands for the prosthesis actuation system on the basis of the sensory information from the sensors embedded into the prosthesis; (3) the sensory subsystem for closing the loop on the amputee by returning the sensation produced by the contact with the manipulated object.

It has been shown that myoelectric control is widely used for controlling electronically-driven hand prostheses, mostly through on/off and proportional approaches. Although these techniques offer the user a practical and reliable way to deal with the prosthesis, the control is unnatural and requires a great mental effort. Moreover, each movement can be activated only in sequence, with a behavior far from the multifunctional capabilities of the human hand.

In order to reduce the patient effort in controlling the prostheses, pattern recognition techniques have been introduced. Over the years, technological improvements made it possible to increase performance of these algorithms, with the drawback of also increasing their complexity. They allow decoding the motion intention with a high level of reliability and relatively low time of response. The complex nature of forearm muscles synergies, the cross talk effect and the displacement of the muscles during contraction are three important drawbacks of pattern recognition-based controllers that limit its usage in clinical setting.

Recently, NMF method have been applied to EMG control, reaching an intuitive, simultaneous and proportional control of multiple DoFs.

Commercial prosthesis typically use classical myoelectric control with on/off or proportional techniques. Pattern recognition and NMF are employed and tested in the research field. The limited number of classes, the high number of EMG electrodes, the necessity of long training/calibration phases, the necessity to simultaneous control multiple DoFs and the variation of signals in relation to arm position are the main liming factors for their employment in the clinical practice. Therefore, future challenges in this area should aim at developing control algorithms robust to variation of arm position and able to simultaneously and independently control multiple DoFs.

Section II.b reports the state of art on the sensorization of the prosthetic hands. Different tactile sensors based on a huge variety of functioning principles have been developed and adopted in the literature for measuring applied forces and slippage. As it can be observed, in the research field there are no examples of hands capable of providing these two kinds of information from sensors embedded in a prosthetic hand, except for the Southampton Hand. However, the embedded slippage sensors, due to their functioning and constructive principles, showed a noisy behavior, in addition to a temperature-dependent output. As far as concerns commercial prostheses, it is worth noticing that there is only one available mechatronic prosthesis on the market, the SensorHand Speed by Ottobock^7^, which rely on tactile sensors mounted only on one of the three fingers of the hand. Thanks to such sensors (SUVA System), grip force can be adjusted so as to avoid slippage events.

It is worth of observing that integration of tactile sensors in artificial hands is not the real limiting factor of hand sensorization. The real challenge is to provide the patients with such information in a reliable way.

The studies analyzed in Sect. II.c provided interesting insights into the mechanisms subtending neural control and sensory feedback restoration. They provided an evidence of the possibility of using efferent neural signals to control a prosthetic hand (Rossini et al., 2010) and afferent neural stimulation to elicit sensory feedback (Ortiz-Catalan et al., 2014a; Raspopovic et al., 2014; Tan et al., 2014), thus closing the amputee in the prosthesis control loop in a stable and repeatable manner for long period, as in the case of the implant of cuff electrodes (Ortiz-Catalan et al., 2014a; Tan et al., 2014).

The four analyzed studies (Rossini et al., 2010; Ortiz-Catalan et al., 2014a; Raspopovic et al., 2014; Tan et al., 2014) have presented different solutions in terms of efferent pathways. The user intentions of movement have been extracted starting from muscular signals in Rossini et al. (2010), Raspopovic et al. (2014). Despite the high level of invasiveness of implanted EMG electrodes, in Ortiz-Catalan et al. (2014a) the controllability of the prosthesis has been demonstrated to improve during the use of epymisial EMG electrodes with respect to superficial electrodes. The control of the prosthesis through implanted electrodes appears to be more robust to limb position and environmental conditions with respect to the use of superficial electrodes.

The use of neural electrodes as in Rossini et al. (2010) permits to implement a natural control, since it allows using the nerve for the execution of motor command as well as for the restitution of sensory feedback. This solution is evidently more invasive than superficial EMG and has been experimentally tested for a limited amount of time (4 weeks). Moreover the use of ENG signals to control prosthetic hand has needed decoding algorithms with a higher computational burden respect to EMG control due to the difficulty to observe and extract neural spikes (Cloutier and Yang, 2013).

On the other hand, on the afferent pathway neural electrodes (cuff or intraneural) have been used to stimulate the nerves and restore the tactile sensory feedback. The implant of intraneural electrodes has the advantages of a higher number of active sites with respect to cuff or FINE electrodes and, more importantly, a higher level of selectivity, thanks to the lower current required to activate nerve fibers and the small groups of fiber involved in stimulation (Saal and Bensmaia, 2015). Indeed, the activation of a large group of afferent fibers through a single cuff electrode, is unnatural and can evoke paresthesia (Tan et al., 2014). As a disadvantage, the level of invasiveness of intraneural electrodes is higher than cuff. Moreover, the experimental validation of intraneural electrodes was limited to a short period of 4 weeks while cuff electrodes were employed for long experimentations of 1–2 years.

The first attempt of establishing a bidirectional control of a prosthesis was performed in 2005 in Dhillon and Horch (2005). In Raspopovic et al. (2014) the bidirectional control of the prosthesis was employed to recognize shape, position and consistence of different object during grasp. In Rossini et al. (2010), Ortiz-Catalan et al. (2014a) the afferent and efferent pathways were tested separately, while in Tan et al. (2014) the restoration of tactile feedback in chronic implants was demonstrated. In Schiefer et al. (2015) an extension of Tan et al. (2014) has been proposed by using afferent information for improving prosthetic hand control.

Notwithstanding the reviewed studies have to be acknowledged for the disruptive innovation they have brought in the prosthetic field, a number of further challenges still need to be faced by the future research studies, e.g., to improve the quality of the stimulation signal and the stimulus temporization on the user force loop in order to convey more natural sensations, in a more selective manner; to increase the number of sensory information returned to the user (such as proprioception, pain, temperature, slippage); to make the user learn how to use this information; enlarge the number of grasps and extend prosthesis capabilities to manage force/tactile information and perform manipulation tasks where contact-no contact transition may occur while handling the objects.

A set of suggestions can be extracted from the literature for each subsystem of the PNS-based prosthetic device (Figure 1), based on the assumption that investigating and replicating the behavior of the biological system can help achieving performance comparable with the natural hand.

The *efferent control system* should satisfy the following suggestions (Zecca et al., 2002; Peerdeman et al., 2011; Scheme and Englehart, 2011):

- To allow the patient to intuitively generate natural movements; in particular, the control should be proportional and able to activate more functions simultaneously (and not in sequence);
- To be robust with respect to arm position variation, alteration of impedance between electrodes and skin, signal variation during daily continuative usage;
- To adapt to the specific state of each subject, thus being able to automatically compensate for behavior changing due to fatigue or sweating;
- To work with the lowest number possible of electrodes, and be blind to electrodes location;
- To require a short training/calibration phase with respect to the effective use of the trained hand;
- To include an alternative feedback to vision, e.g., on force/tactile, in order to rely less on visual attention and close the control loop on the patient;
- To return natural feelings of the interaction with the object through force or tactile feedback.
- To avoid incorrect classification and undesired movements by means of an on-line examination and processing of the ENG signals (Rossini et al., 2010).
- To be computationally efficient and embeddable on the prosthetic device;
- To guarantee real-time functioning (delay lower than 125 ms, Farrel and Weir, 2007).

**Figure 4:**
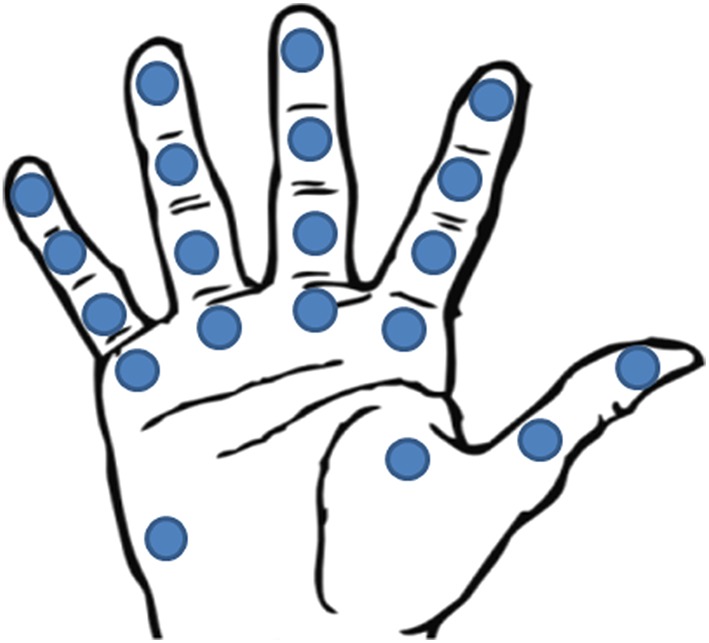
**Location of the 20 FSR sensors in Kargov et al. (2004)**.

The *force/tactile sensory subsystem* should meet the suggestions listed below:

- To provide information such as: (i) actual contact with the object; (ii) applied forces to the object; (iii) detection of object superficial properties; (iv) object slippage detection.
- To have good static response and satisfying dynamic response, in order to measure forces and detect slippage.
- To measure for each finger a force range of [0.15–10 N], since manipulation forces exerted by each finger of the human hand are comprised between 0.15 and 0.9 N (Dahiya et al., 2009) and forces up to 10 N are applicable during grasping.
- To apply with the prosthesis forces at least up to 70 N, since the upper force limit of human hand performing “activities of daily living” is about 68 N (Belter et al., 2013).
- To guarantee force resolution lower than 0.5 N, being 0.5 N the haptic resolution for human fingers (Shimoga, 1993); however, deeper resolution are possible with the existing technologies.
- To ensure sensor bandwidth ranging from 1 KHz up to 1.5 KHz, being the biggest frequency detectable by mechanoreceptors of human hand (Pacinian Corpuscles, 500–700 Hz). Narrower bandwidths are however acceptable, depending on the physical principle exploited by the tactile sensors mounted on the prosthesis.
- To optimize the sensor spatial density on the prosthetic hand (Figure 4). In order to mimic the natural hand, it should be considered that spatial density is not the same all over the hand; it is maximum on the fingertip and decreases along the finger toward proximal direction, coming to be minimum in the palm. Meissner Corpuscles reach densities of 140 units/cm^2^, while Merkel Disks density is less or more the half, i.e., about 70 units/cm^2^. The resolution is very high, less than 2 mm. However, different spatial distribution of the sensors can be considered, taking into account the structure of the prosthetic hand and, mainly, the grasping tasks it is able to perform. A good compromise between grasping capabilities and sensor spatial distribution seems to be offered by the sensorization of 20 different areas of the hand (Figure 4), corresponding to fingertips, finger phalanges, finger MCP joints and thenar and hypothenar eminence (Kargov et al., 2004; Kondo et al., 2008).

The following main suggestions should be summarized for the *afferent pathway of the interfacing subsystem*:

- Maximization of the number of active sites on the electrode in order to: (i) raise the type of elicited sensations; (ii) increase sensitive areas of the hand; (iii) improve the specificity of these areas and help its identification.
- In case of electrodes with few active sites, a high number of electrodes is needed to assure a good spatial resolution and a good selectivity (Navarro et al., 2005).
- Transversal insertion for intraneural electrodes, in order to maximize selectivity.
- The neural interface has to guarantee stability for all implant lifetime and biocompatibility.
- Stimulation parameters have to be identified in relation to the adopted electrode as revealed from human experimentation results (Section Neural Control and Sensory Feedback Restoration).

## Conclusions and future perspectives

This paper has provided an overview of the main advancements reached in prosthetic field, with particular attention to prosthetic hands controlled via PNS-based interfaces. Technical features (control strategies, sensory system) and aspects regarding human experimentations (neural implants for sensory feedback restoration, neuroplasticity evaluation after amputation) have been debated and a set of main suggestions of the ideal bidirectional hand prostheses (Figure 1) have been presented.

This paper reveals the necessity to improve the hand control and the way for exchanging sensory information between the environment and the user. The employment of the proposed suggestions in the clinical practice mainly depends on the stability and reliability of the developed hardware and software solutions over long periods of time.

The investigation of the requirements that the whole prosthetic system, so as each subsystem, should fulfill to overcome the current limitations in this field has shown that the prosthetic system of the future should: (i) embed a new generation of control algorithms, able to manage more sensory information (tactile perception, proprioception, pain and temperature) (Farina and Aszmann, 2014) and increase hand grasping and manipulation capabilities, (ii) be equipped with a sensory system able to provide reliable information to the hand control, (iii) guarantee a natural control of the prosthesis by establishing a bidirectional communication via neural interfaces, (iv) guarantee stability of the restored tactile sensation for chronic implants.

## Author contributions

AC and FC designed the paper, analyzed the literature and wrote the paper; RB and AB analyzed the literature about prosthetic hand control and wrote the corresponding paragraph, RR analyzed the literature about hand sensorization and wrote the corresponding paragraph, RS, AD collaborated during the literature analysis and revised the paper; GD, FR, and VD wrote the paragraph regarding anatomy and physiology background; EG and LZ designed the paper and supervised the writing. All the authors read and approved the manuscript.

## Funding

This work was supported partly by the National Institute for Insurance against Accidents at Work (INAIL) with the PPR 2 project (CUP: E58C13000990001), partly by the Italian Ministry of Instruction, University and Research with PRIN HANDBOT project (CUP: B81J12002680008) and partly by the Italian Ministry of Health with NEMESIS project (CUP: C81J12000380001).

### Conflict of interest statement

The authors declare that the research was conducted in the absence of any commercial or financial relationships that could be construed as a potential conflict of interest.

## Footnotes

^1^http://www.salute.gov.it/ricoveriOspedalieri/ric_informazioni/default.jsp

^2^http://www.touchbionics.com/

^3^http://www.living-with-michelangelo.com/home/

^4^http://www.bebionic.com

^5^http://main.biorobotics.it/menu-projects/158-handbot

^6^Free functioning muscle transfer is a surgical technique based on microvascular anastomosis and fascicular nerve repair (Battyeet et al., 1955), used to restore upper limb functions in patients with functional deficits (Popov, 1965).

^7^COAPT Complete Control, 2014 Available: https://www.coaptengineering.com/

^8^http://www.computerhistory.org/collections/catalog/102693567

^9^http://www.ottobock.com

^10^http://www.prensilia.com/

^11^http://www.dorset-ortho.com/downloads/SensorHand%20Speed.pdf
